# Supplementary material for: Design of a symmetry-broken tetrahedral protein cage by a method of internal steric occlusion
Source: bioRxiv. 2023 Nov 9:2023.11.08.566319. Preprint. [Version 1] doi: 10.1101/2023.11.08.566319 (PMC10659388; doi:10.1101/2023.11.08.566319)
Supplement: Supplement 1 [file media-1.pdf]

Supplementary Material for

Gladkov, *et al.*
"Design of a symmetry-broken tetrahedral protein cage by a method of internal steric occlusion"

Supplementary Figure:

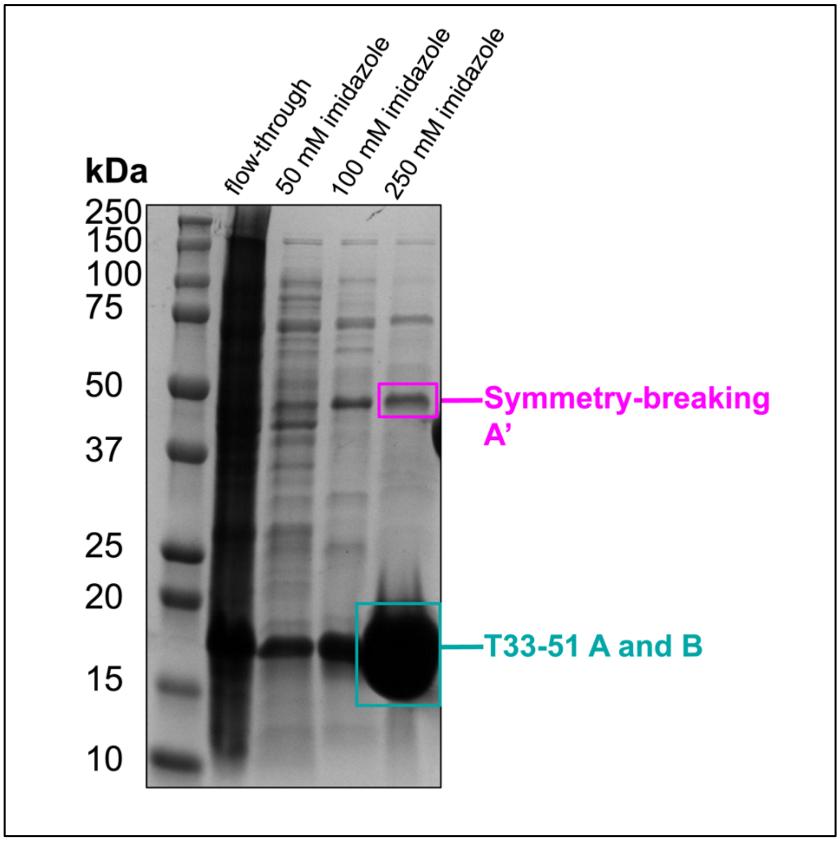

Figure S1. SDS-PAGE of immobilized nickel affinity chromatography fractions from the purification of symmetry-broken cage eluted with a linear imidazole gradient from 50 mM to 250 mM.

Supplementary Text:

Protein sequences:

T33-51 A	MSNEEVWKDDPIIEANGTLDELTSFIGEAKHYVDEEMKGILEEIQNDIY<br>KIMGEIGSKGKIEGISEERIKWLAGLIERYSEMVNKLSFVLPGGTLESA<br>KLDVCRTIARRAERKVATVLREFGIGTLAAIYLALLSRLLFLLARVIEIEK<br>NKL
T33-51 A’	MSDSEVNQEAKPEVKPEVKPETHINLKVSDGSSEIFFKIKKTTPLRRL<br>MEAFAKRQGKEMDSLRFLYDGIRIQADQTPEDLDMEGGSSGSNEEV<br>WKDDPIIEANGTLDELTSFIGEAKHYVDEEMKGILEEIQNDIYKIMGEI<br>GSKGKIEGISEERIKWLAGLIERYSEMVNKLSFVLPGGTLESAKLDVC<br>RTIARRAERKVATVLREFGIGTLAAIYLALLSRLLFLLARVIEIEKNKLGP<br>GSDLGKKLLEAARAGQDDEVRILMANGADVNAADDVGVTPLHLAAQ<br>RGHLEIVEVLLKYGADVNAADLWGQTPLHLAATAGHLEIVEVLLKNG<br>ADVNARDNIGHTPLHLAAWAGHLEIVEVLLKYGADVNAQDKFGKTPF<br>DLAIDNGNEDIAEVLQKAA
T33-51 B	MFTRRGDQGETDLANRARVGKDSPVVEVQGTIDELNSFIGYALVLSR<br>WDDIRNDLFRIQNDLFVLGEDVSTGGKGRTVTMDMIIYLIKRAVEMKA<br>EIGKIELFVVPGGSVESASLHMARAVSRRLERRIKAASELTEINANVLL<br>YANMLSNILFMHALISNKRLNIPEKIWSIHRVSLEHHHHHH
